# A mixed-methods study of the awareness and functionality of sexual and reproductive health services among persons with disability in Ghana

**DOI:** 10.1186/s12978-023-01700-1

**Published:** 2023-10-31

**Authors:** Abdul-Aziz Seidu, Bunmi S. Malau-Aduli, Kristin McBain-Rigg, Aduli E. O. Malau-Aduli, Theophilus I. Emeto

**Affiliations:** 1 Public Health and Tropical Medicine, College of Public Health, Medical and Veterinary Sciences, James Cook University, Townsville, QLD 4811 Australia; 2 Department of Population and Health, University of Cape Coast, P.O. Box UC 182, Cape Coast, Ghana; 3 College of Medicine and Dentistry, James Cook University, Townsville, QLD 4811 Australia; 4 School of Medicine and Public Health, University of Newcastle, Newcastle, NSW 2308 Australia; 5 School of Environmental and Life Sciences, The University of Newcastle, Newcastle, NSW 2308 Australia; 6 World Health Organization Collaborating Center for Vector-Borne and Neglected Tropical Diseases, James Cook University, Townsville, QLD 4811 Australia

**Keywords:** Awareness, Disability, Functionality, Ghana, HIV/AIDS, Knowledge, Sexual and reproductive health, STIs

## Abstract

**Background:**

Persons with disabilities (PwDs) face barriers in accessing sexual and reproductive health (SRH) services due to lack of knowledge and awareness, stigma and discrimination, and inadequate service provision. This study aimed to examine the determinants of SRH knowledge and awareness among PwDs in Ghana, and to explore their perceptions of the functionality of SRH services.

**Methods:**

A sequential explanatory mixed-methods study design was used to collect and analyse quantitative (n = 402) and qualitative (n = 37) data from PwDs in two districts in Ghana. Quantitative data were analysed using descriptive and inferential statistics, while qualitative data was analysed thematically.

**Results:**

Most of the participants had high awareness of SRH (94.3%), sexually transmitted infections (STIs) (92.5%) and HIV/AIDS (97.0%). Employment status was positively associated with SRH awareness [aOR = 1.62; 95% CI = 1.02, 2.59]. Disability type was a significant predictor of STI [aOR = 2.02; 95% CI = 1.39, 2.94] and HIV/AIDS [aOR = 2.32; 95% CI = 1.21, 4.44] awareness, with the visually impaired having higher odds than the physically disabled. Age group was also a significant predictor of STI awareness, with older respondents having higher odds than younger ones [aOR = 1.76; CI = 1.01,3.05 for 30–39 years; aOR = 2.48; CI = 1.22, 5.05 for 40–49 years]. The qualitative findings revealed four main themes: conceptualisation of SRH, active engagement in SRH information seeking, tensions between knowledge and religious beliefs and perceived utility of SRH services.

**Conclusion:**

Despite the high levels of SRH knowledge and awareness among PwDs, there are significant gaps and challenges related to disability type, age group, misconceptions, beliefs, and service non-functionality that limit the utility of SRH services. The findings call for tailored education to reduce misconceptions and put in pragmatic steps to deliver quality SRH services and information to PwDs. Further research is needed to assess the sexual lives of PwDs and explore the perspectives of all relevant stakeholders, including service providers and policymakers on how to enhance SRH outcomes for PwDs in Ghana.

**Supplementary Information:**

The online version contains supplementary material available at 10.1186/s12978-023-01700-1.

## Plain language summary

People with disability often encounter barriers in access sexual and reproductive health (SRH) services. These challenges arise from a lack of understanding and awareness about these services, stigma, and discrimination as well as unavailability of these services. This study sought to assess the extent of SRH knowledge and awareness among persons with disabilities (PwDs) in Ghana and to investigate their perceptions of the functionality of SRH services. To accomplish this, a study design that combined both quantitative and qualitative research methods was employed. The study collected and analysed data from 402 PwDs in two districts of Ghana. The quantitative data were subjected to descriptive and inferential statistical analyses, whereas the qualitative data, were thematically analysed. The findings indicated that a substantial number of participants demonstrated high awareness of SRH, including sexually transmitted infections (STIs) and HIV/AIDS. The participants' employment status was positively associated with their level of SRH awareness. Additionally, the type of disability played a significant role in predicting awareness of STIs and HIV/AIDS. Visually impaired individuals exhibited a greater likelihood of awareness compared to those with physical disabilities. Age also influenced STI awareness, with older respondents showing higher levels of awareness than their younger counterparts. Qualitative analyses unveiled four main themes: how SRH is conceptualised, the active seeking of SRH information, the tension between knowledge and religious beliefs, and the perceived usefulness of SRH services. The study concluded that PwDs have a high level of SRH knowledge and awareness. However, it identified gaps and challenges that stem from differences in disability type, age group, misconceptions, religious beliefs, and the effectiveness of the services provided. To address these issues, the study recommends tailored educational interventions to meet the unique needs and concerns of PwDs in relation to SRH. Furthermore, enhancing the quality and accessibility of SRH services for this population is suggested. The study underscores the need for further research on the sexual lives of PwDs and the views of stakeholders on how to enhance their SRH outcomes are needed.

**Supplementary Information:**

The online version contains supplementary material available at 10.1186/s12978-023-01700-1.

## Introduction

According to the World Health Organization (WHO), 16% of the global population, or about one billion people, have some form of disability, and 80% of them live in low-and middle-income countries [1]. In Africa, the disability prevalence is estimated at 10%, which corresponds to 60–80 million, but this figure may be higher in regions with high levels of poverty [2]. Ghana has a disability prevalence of 8%, with the Ashanti region having the highest prevalence rate of 17% [3]. The number of people with disabilities (PwDs) is expected to increase due to factors such as ageing, accidents, and chronic health conditions, including those related to sexual and reproductive health (SRH) outcomes, such as sexually transmitted infections (STIs), Human Immunodeficiency Virus (HIV) [4], and Acquired Immunodeficiency Syndrome (AIDS) [5].

SRH is defined by the WHO as “a state of complete physical, emotional, mental, and social wellbeing in relation to all aspects of sexuality and reproduction, not merely the absence of disease, dysfunction, or infirmity” [6]. SRH is a fundamental aspect of health and wellbeing and a key objective of the sustainable development goal (SDG) 3.7–﻿which aims to “ensure universal access to SRH services, including family planning, information and education, and the integration of reproductive health into national strategies and programmes” by 2030 [6]. SRH encompasses four main dimensions: sexual health, sexual rights, reproductive health, and reproductive rights [6]. Sexual health refers to the prevention and treatment of STIs and other sexual problems [7], while sexual rights include the right to sexual education, the freedom to choose a partner, the right to consensual sex, and freedom from sexual abuse [8]. Reproductive health involves the ability and capacity to learn about the reproductive system, contraception, and safe abortion services [9, 10], while reproductive rights entail the recognition and respect of individuals’ autonomy and decision-making regarding their reproductive lives [11].

STIs and HIV/AIDS are major global public health challenges that affect millions of people every year [12–14]. Therefore, access to accurate and comprehensive information on SRH is essential for PwDs to protect themselves from infections, enjoy healthy relationships, exercise their sexual and reproductive rights, and achieve optimal wellbeing [15]. Access to accurate and comprehensive information on HIV/AIDS and its transmission is essential to prevent and reduce the spread of the infection. However, sub-Saharan Africa (SSA) has a high burden of STIs and HIV/AIDS, partly due to low levels of knowledge and awareness among the population [16]. Therefore, improving SRH knowledge and awareness is critical for vulnerable groups such as PwDs, who face multiple barriers to accessing SRH information and services [17]. Previous studies have reported inconsistent findings on the level of SRH knowledge and awareness among PwDs in different settings, depending on the indicators used and the population studied. The reported prevalence ranges from 46% to 97.2%. For instance, studies in Ethiopia have found that knowledge on family planning methods varied from 46% to 97.2%, while knowledge on HIV/AIDS and STIs ranged from 46.9% to 79.8% among PwDs [18–21]. Similarly, studies in Turkey and Uganda have reported that 70.9% and 93% of PwDs were informed about STIs and HIV transmission respectively [18, 22]. In Ghana, a study by Obasi et al.[23] reported 67.1% of knowledge on SRH. Some of the factors associated with SRH awareness and knowledge among PwDs are age, sex, marital status, employment status, religion, and sources of information [9, 17, 19, 20, 23–25].

Several policies and interventions have been introduced in Ghana to enhance the provision and accessibility of SRH services and information to the general population. These include the National Health Insurance Scheme [26], the National Health Policy [27], the Disability Act 715 [28], the Adolescent Health Service Policy and Strategy [29] and the Free Maternal Healthcare Policies [30, 31]. However, there is limited evidence on how these policies and interventions have affected the awareness and functionality of the SRH services for PwDs [9, 25, 32]. Moreover, there is a dearth of studies on the level and determinants of SRH knowledge and awareness among PwDs in Ghana. Most of the existing studies have either focused on a narrow aspect of SRH such as abortion [32] or contraceptive methods [9] or targeted specific groups of PwDs, such as those with physical disabilities [33] or visual impairments [34]. To address this gap, this study employs a mixed-methods approach to assess the awareness and knowledge of SRH services among PwDs (visually impaired and physically disabled) and their perceptions on the functionality of SRH services and interventions in the Ashanti region of Ghana. The findings of this study will provide useful insights for stakeholders to design and implement tailored interventions to improve the SRH knowledge and outcomes among PwDs.

### Theoretical framework

The current study is an aspect of a larger study that uses the health outcomes model [35–37] to assess the *"Impact of health policies and interventions on the sexual and reproductive health outcomes among persons with disabilities in Ghana”.* The framework describes how system characteristics, interventions, and client/individual characteristics all affect health outcomes or behaviours [37]. Client characteristics encompass demographic and disability related factors. Interventions encompass both direct and indirect measures, as well as initiatives by government, healthcare professionals, and Non-Governmental Organizations aimed at enhancing the health of PwDs. System characteristics pertain to the organization of systems, such as hospitals or healthcare provider networks, and their interactions, which collectively shape an individual's health [37]. A diagram showing the various components of the model and its detailed description have previously been published [38, 39]. This current study focused specifically on three aspects of the framework: system characteristics, client characteristics, and interventions. The three aspects of the framework were chosen due to the current study’s objective of assessing the awareness and perceptions of PwDs regarding the functionality of SRH services. The individual or client aspect of the framework plays a significant role in shaping PwDs' awareness and perceptions of SRH, while the nature of the health delivery system also impacts the flow of SRH information to PwDs. The awareness and perception of SRH functionality are influenced by the interactions between population characteristics and the healthcare system [34, 40, 41]. Therefore, access to SRH information is achieved when healthcare resources align with individual healthcare needs and are appropriately delivered by HPs [34]. This framework is considered ideal for the present study, as it has been validated [36] and utilised across various settings and populations [38, 39, 42].

## Methods

### Ethics approval

Ethical clearance was obtained from three institutional review boards: the Ghana Health Service (GHS) Ethics Review/committees (GHS-ERC: 005–0621), the Komfo Anokye Teaching Hospital (KATH) (KATH-IRB/RR/101/21), and the James Cook University (JCU) Human Ethics Committee (H8531). The study also received approval from the Regional Health Directorate in Kumasi and the Offinso North District Health Directorate in Akumadan. Additionally, the study obtained both written and verbal consent from the leaders of two disability groups (Ghana Association of the Blind and the Ghana Society of the Physically Disabled) in the Kumasi Metropolis and Offinso North District. Furthermore, the study ensured the anonymity and confidentiality of the participants. Prior to administering the study instrument, a document explaining the study objectives, anonymity and confidentiality, and benefits of participation was provided to the participants. They were also informed that their involvement was voluntary and that they could withdraw from the project at any time without any repercussions. They were also assured that their data would be used only for academic purposes and that their identities would not be disclosed to anyone. Both written and verbal informed consents were obtained from all participants. In addition to ethical approval and informed consent, all methods adhered to the Declaration of Helsinki's ethical principles for conducting human research.

### Study design

**Fig. 1:**
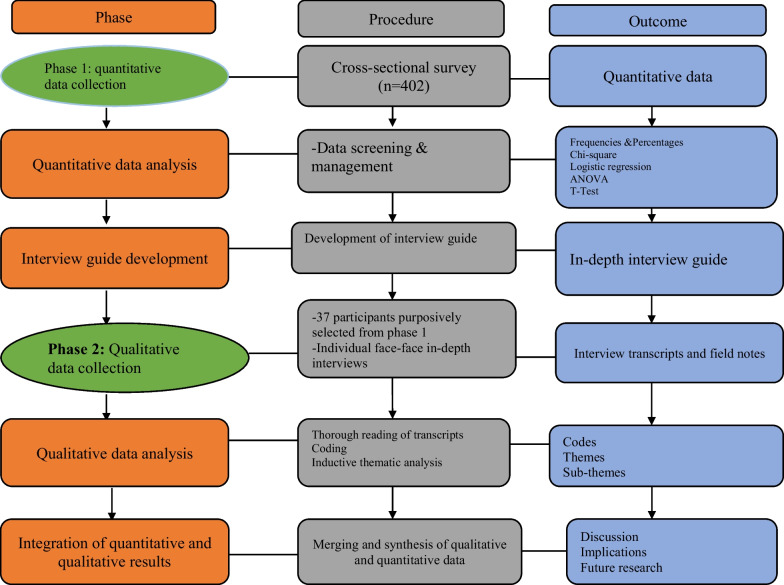
Sequential explanatory mixed-methods [43]

The study adopted a sequential explanatory mixed-methods design [43] (Fig. 1) using a pragmatic paradigm to collect data from PwDs in the Ashanti Region. “This design consists of two distinct phases: quantitative followed by qualitative. ﻿Its advantages include straightforwardness and opportunities for the exploration of the quantitative results in more detail” ([43], p5). The quantitative phase used a cross-sectional study design with surveys to measure the awareness, knowledge, and perception of SRH services functionality among PwDs, while the qualitative phase used, a descriptive phenomenological design [44] to explore the lived experiences and perceptions of PwDs on the knowledge and functionality of SRH services and interventions. The participants included those with physical disabilities and those with visual impairments. The last question in the survey (quantitative phase) was used to identify and recruit voluntary participants for the interviews (qualitative phase). This allowed for a deeper understanding of some of the issues raised in the quantitative phase.

### Study setting and target population

According to the 2021 Population and Housing Census report, the Ashanti Region had the highest proportion of PwDs in Ghana (17.3%) [3]. The study focused on those with visual/seeing (4%) and physical impairment/walking (3.6%) which were the most common types of disabilities in this area, with more PwDs living in urban (9.5%) than rural (6.5%) areas. Details of the study area have been published elsewhere [38]. The inclusion criteria for participant selection were (a) being 18 years or older, and (b) having either physical or visual impairment. The exclusion criteria were (a) being under 18 years old, (b) having multiple disabilities, and (c) having a disability other than physical or visual impairment.

### Recruitment and training of research assistants

Four experienced research assistants (RAs) from the Department of Population and Health, University of Cape Coast, Cape Coast and the Department of Health Promotion and Disability Studies, School of Public Health, Kwame Nkrumah University of Science and Technology, Kumasi, Ghana were purposively recruited. They held bachelor and master degrees in the field of Population and Health, Disability and Rehabilitation as well as Geography. They had knowledge of disability issues and survey data collection. They underwent a five-day training using a designed training manual. The principal investigator (AS) trained them on the context, tool contents, and protocols for conducting successful interviews, including ethics and informed consent.

### Phase one: quantitative phase

#### Sample size and sampling

The sample size for the quantitative phase was calculated using the formula by Lwanga, Lemeshow, and WHO [45], which yielded 402 PwDs. The sampling frame was obtained from the list of PwDs provided by the group leaders of different disability categories. The participants were selected from the list using a systematic sampling technique [46] and contacted during their meetings to invite them to participate in the survey. Details of the sampling procedure have been published elsewhere [39].

#### Survey instrument development and data collection

The questionnaire (Additional file 1: Appendix S1) was developed based on a previously validated tool [47] and a review of the literature [17, 19, 20, 23, 24, 33]. The questionnaire was used to collect the primary data for the quantitative phase. RAs administered the questionnaires to the respondents face-to-face. The RAs read and interpreted survey questions to respondents. Once responses were given, assistants recorded accordingly. The data collection took place between January 10 and April 24, 2022. Before the data collection, the questionnaire was pretested among 30 PwDs in Nkawie and Nkenkaasu in the Ashanti region and all the identified ambiguities were resolved before the questionnaire was administered to all participants.

#### Variables

The dependent variables were knowledge on SRH services (SRH, STIs and HIV/AIDS), which were binary and functionality of SRH services. Functionality was how SRH services are meeting PwD’s needs. The respondents were asked to respond to a list of seven different statements on a 4-point Likert Scale The scale had a reliability score of 0.8049. The independent variables were the socio-demographic characteristics and disability-related factors (Additional file 1: Appendix S1).

#### Statistical analyses

Descriptive statistics, such as frequencies and percentages were used to describe the socio-demographic characteristics of the participants. Complementary log–log regression analysis was also employed to assess the factors associated with PwDs awareness of SRH, STIs and HIV/AIDS. The complementary log–log regression analysis was adopted because it is suitable for binary outcomes with uneven distributions, whereas Probit or Logit functions could produce biased estimates [48]. T-test and one-way analysis of variance (ANOVA) were used to assess the relationship between background characteristics and perception of functionality of SRH services score. Regarding the perception of functionality of SRH services, a mean score was computed from the responses and compared with a criterion mean of 2.5 ([1 + 2 + 3 + 4]/4). Mean scores greater than 2.5 suggested that the specific form of SRH is unfunctional whereas mean scores below 2.5 reflected functionality of the SRH service. For the overall functionality score, a total mean score was computed from the responses and compared with a criterion mean of 14. Mean scores greater than 14 indicated unfunctional SRH services, whereas mean scores below 14 reflected functional SRH services. The level of statistical significance was set at p < 0.05. All the statistical analyses were performed using Stata version 14 (StataCorp LP, College Station, TX, USA) [49].This study followed the strengthening the reporting of observational studies in epidemiology (STROBE) checklist and explanation (Additional file 2: Appendix S2) [50].

### Phase two: qualitative phase

The aim of the second phase of the study was to delve deeper into the issues that emerged from the first phase. A semi-structured interview guide (Additional file 3: Appendix S3) was designed to elicit rich qualitative data from the participants and to gain a better understanding of their SRH knowledge and perceptions of the functionality of SRH services. A purposive sampling technique was employed to select 37 PwDs from the two study sites, ensuring a balance of gender/sex and type of impairment. The lead author (AS) and a female RA conducted face-to-face interviews with the participants at private and convenient locations, such as their homes or meeting venues. A pilot test of the interview guide was conducted with four PwDs from Nkawie (2) and Nkenkaasu (2) in the Ashanti region to assess its clarity and comprehensibility. The interviews were conducted between May 5 and July 11, 2022. Verbal and written consent were obtained from the participants and the interview duration ranged from 45 to 70 min, with an average of 57 min. Data saturation was reached after interviewing the 35th participant, with no new information emerging at this point [51]. However, two more interviews were conducted with interested individuals to ensure that no valuable information was missed. Therefore, data collection was stopped after interviewing the 37th participant.

### Qualitative data analysis

All the audio recordings were transcribed verbatim by AS and the RA and checked for accuracy. The transcripts were anonymised to protect the participants’ identity and imported into NVivo version 12 (QSR International Pty Ltd; Version 12). Braun and Clarke's [52] six step-thematic analysis approach was followed to generate data-driven themes. First, AS read the transcripts thoroughly to familiarise with the data, under the supervision of KM-R and TIE through regular meetings. Second, AS, KM-R and TIE coded the data and collated the codes into key topics. Third, AS, KM-R and TIE grouped the codes by comparing them and identifying emerging themes. Fourth, KM-R, BM-A and TIE reviewed the themes and checked their interrelationships and relevance. Fifth, the themes were defined, refined, and confirmed by the Team. Illustrative quotes were presented verbatim, and characterised with the demographic information of the participants, including the disability type, location, gender/sex, and age of the interviewee [for example—Visually Impaired, Kumasi, Female, 21 years]. The qualitative phase of the research was reported following the Consolidated criteria for reporting qualitative research (COREQ) [53] (Additional file 4: Appendix S4). The trustworthiness of the qualitative data was ensured by following Lincoln and Guba [54] key strategies.

## Results

### Phase one: quantitative findings

#### Socio-demographic characteristics of respondents

The socio-demographic characteristics of the 402 respondents are summarised in Table 1. Majority of the respondents had visual impairment (57.7%) and were male (51.5%). Most of the respondents lived in urban areas (80%), belonged to the Akan ethnic group (82%), followed Christianity (88.6%), and subscribed to the National Health Insurance Scheme (NHIS) (96.8%). About a third of the respondents were aged 60 years or older (30%), had senior high school/tertiary education (34%), and about half of them were employed (50%) and married (44%).

**Table 1:** Socio-demographic characteristics of respondents

Variable	Frequency	Percentage
Disability type		
Physically disabled	170	42.3
Visually impaired	232	57.7
Age (years)		
18–29	46	11.4
30–39	79	19.7
40–49	73	18.2
50–59	89	22.1
60 and above	115	28.6
Sex		
Female	195	48.5
Male	207	51.5
Residence		
Kumasi metropolis	323	80.4
Offinso north	79	19.7
Level of education		
No formal education	68	16.9
Primary	66	16.4
Junior High School	130	32.3
Senior High School/Tertiary	138	34.3
Religious affiliation		
Christian	356	88.6
Non-Christian	46	11.4
Marital status		
Never married	97	24.1
Married	178	44.3
Separated/Widowed/Divorced	127	31.6
Ethnicity		
Akan	330	82.1
Non-Akan	72	17.9
Employment		
Not working	199	49.5
Working	203	50.5
Income (GHC)		
0–99	203	50.50
100–299	102	25.37
300 +	97	24.13
NHIS Subscription		
No	13	3.2
Yes	389	96.8
Duration to the nearest health facility		
Less than 30 min	133	33.1
30–59 min	192	47.8
60 min and above	77	19.2

*NHIS* National Health Insurance, *GHC* Ghana Cedis

#### Functionality of sexual and reproductive health services

﻿In Table 2, the overall functionality score (mean = 15 ± 4.9) indicates that SRH services were not functional to meet the needs of PwDs. Table 3 shows that the visually impaired perceived SRH services to be less functional compared to the physically disabled (16.6 ± 5.0, p < 0.001). PwDs who had higher levels of education perceived SRH services to be less functional compared to those with no formal education (16.7 ± 5.3, p < 0.001). PwDs who earned GHC300 or more perceived SRH services to be less functional compared to those who earned GHC100-299 (14.6 ± 4.9, *p* < 0.001) (Table 3).

**Table 2:** Perceptions of functionality of sexual and reproductive health services

Statement	Obs	Mean	SD	Range
				Min–Max
Information provision by the government, health officials and NGOs on SRH to PwDs	402	1.8	0.98	1–4
Provision and delivery of reproductive health services to PwDs	402	1.8	0.97	1–4
Comprehensive sexuality education to PwDs	402	2.6	1.0	1–4
PwDs youth empowerment on SRH issues	402	2.6	1.0	1–4
Training of healthcare providers on how to render SRH services to PwDs	402	2.0	1.1	1–4
Education of community members on SRH issues in relation to PwDs	402	2.4	1.1	1–4
Testing and treatment of STIs for PwDs	402	2.0	1.0	1–4
Overall functionality score	402	15.1	4.9	7–28

*Obs*  Observation, *SD* Standard deviation, *min* Minimum, *Max* Maximum, *PwDs* Persons with disabilities, *SRH* Sexual and reproductive health, *NGOs* Non-Governmental organisations

**Table 3:** Background characteristics and knowledge and perceptions about the functionality of sexual and reproductive health services

Variable	Knowledge about SRH			Perception on SRH functionality	
	Sexual and reproductive health	Sexually transmitted infections	HIV/AIDS		
				Mean	SD
	379(94.3%)	372(92.5%)	390 (97.0%)		
Disability type	p = 0.752	p < 0.001	p = 0.005^+^	p≤0.001^a^	
Physically challenged	94.7	87.1	94.1	13.1	3.8
Visually impaired	94.0	96.5	99.1	16.6	5.0
Age (Years)	p=0.242	p=0.073^+^	p = 0.013^+^	p=0.565^a^	
18–29	89.1	82.6	89.1	15.6	4.9
30–39	94.9	92.4	97.5	14.9	4.7
40–49	98.6	97.3	98.6	14.5	4.5
50–59	93.7	94.4	100.0	15.0	5.0
60 and above	93.9	92.2	96.5	15.6	5.0
Sex	p=0.222	p=0.865	p = 0.248^+^	p=0.128^a^	
Female	92.8	92.3	95.9	14.8	4.6
Male	95.6	92.8	98.1	15.5	5.1
Residence	p = 1.000^+^	p = 0.960	p = 0.016	p=0.186^a^	
Kumasi Metro	94.1	92.6	98.1	15.0	5.0
Offinso North	94.9	92.4	92.4	15.8	4.4
Level of education	p=0.075	p=0.183^+^	p = 0.042^+^	p< 0.001^a^	
No formal education	88.2	89.7	95.6	14.9	4.6
Primary	92.4	87.9	92.4	14.3	4.6
Junior High School	96.9	93.1	99.2	14.0	4.1
Senior High School/Tertiary	95.7	95.7	97.8	16.7	5.3
Religious affiliation	p = 0.318	p = 0.367	p = 1.00^+^	p=0.752^a^	
Christian	94.7	93.0	96.9	15.1	4.8
Non-Christian	91.3	89.1	97.8	15.3	5.2
Marital status	p = 0.055^+^	p = 0.221	p = 0.605^+^	p=0.752^a^	
Never married	90.7	88.7	95.9	15.2	4.8
Married	97.2	94.4	97.8	15.0	5.1
Separated/Widowed/Divorced	92.9	92.9	96.9	15.3	4.7
Ethnicity	p = 1.00^+^	p = 0.073	p = 1.00^+^	p=0.429^a^	
Akan	94.2	93.6	97.0	15.2	4.9
Non-Akan	94.4	87.5	97.2	14.7	4.7
Employment	p = 0.004	p = 0.415	p = 0.572	p = 0.355^a^	
Not working	90.9	91.5	96.5	15.4	5.1
Working	97.5	93.6	97.5	14.9	4.6
Income (GHC)	p = 0.022^+^	p = 0.852	p = 0.474^+^	p ≤ 0.001^a^	
0–99	91.1	91.6	96.1	16.1	5.0
100–299	97.1	93.1	97.1	13.6	4.0
300^+^	97.9	93.8	99.0	14.6	4.9
NHIS subscription	p = 0.166^+^	p = 0.252^+^	p = 1.000^+^	p = 0.394^a^	
No	84.62	84.62	100.00	14	4.5
Yes	94.60	92.80	96.92	15.2	4.9
Duration to the nearest health facility	p = 0.685	p = 0.168	p = 0.438^+^	p = 0.328^a^	
Less than 30 min	94.7	91.7	95.5	15.6	4.6
30–59 min	94.8	94.8	97.9	14.8	4.7
60 and above minutes	92.2	88.3	97.4	15.3	5.8

P-values are from Chi-square test; +  = Fishers exact; *GHC* Ghana Cedis, *SD* Standard Deviation, ^a^p-values from ANOVA/t-test

#### Knowledge and awareness about sexual and reproductive health

The participants generally demonstrated high levels of awareness of SRH (94.3%), STIs (92.5%) and HIV/AIDS (97.0%) (Table 3). The Chi-square analysis revealed significant associations between employment (p = 0.004) and income (p = 0.022) and the level of awareness on SRH with those employed and high income being more knowledgeable respectively. Similarly, significant associations were found between disability type and knowledge of STIs (p < 0.001) with the visually impaired having high knowledge. The level of knowledge of HIV/AIDS was also significantly associated with disability type (p = 0.005), age (p = 0.013), residence (p = 0.016) and level of education (p = 0.042) with the visually impaired, those aged 18–29 years and those with higher education more knowledgeable (see Table 3).

#### Importance, sources of information on sexual and reproductive health and signs, symptoms, and methods to prevent STIs

**Fig. 2:**
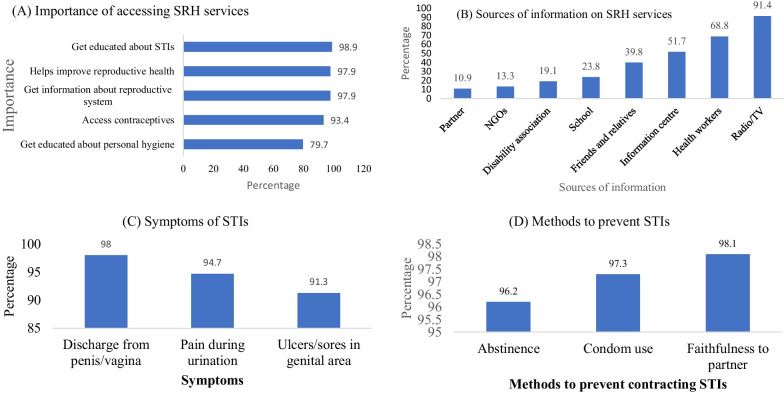
Importance, sources of information on SRH and signs, symptoms, and methods to prevent STIs

Figure 2A illustrates the perceived importance of accessing SRH information and services. The most reported reasons were getting educated about STIs (98.9%), improving reproductive health (97.9%) and obtaining information about reproductive system (97.9%). Almost 80% also reported getting educated about personal hygiene (79.7%). Radio/TV was the most prevalent source of information on SRH, reported by 91.4% of the respondents. Health workers and information centre were also reported by 68.8% and 51.7% respectively. Only 10.9% of the participants reported receiving SRH information from their partner (Fig. 2B). Figure 2C presents the knowledge of the signs and symptoms of STIs among the participants. The majority indicated discharge from penis/vagina (98%)), pain during urination (94.7%) and ulcers/sores in the genital area (91.3%). Most of the respondents indicated faithfulness to partner (96.5%), condom use (95%) and abstinence (94.8%) as the main methods to prevent STIs (Fig. 2D).

#### Factors associated with awareness on sexual and reproductive health, sexually transmitted infections, and HIV/AIDS

Table 4 shows the factors associated with SRH knowledge among the participants. Those who were employed had higher odds of hearing about SRH [aOR = 1.62; 95%CI = 1.02,2.59] compared to those who were unemployed. The results further showed that the visually impaired had higher odds of hearing about STIs [aOR = 2.02; 95% CI = 1.39, 2.94] and HIV/AIDS [aOR = 2.32;95% CI = 1.21,4.44] compared to the physically disabled. PwDs aged 30–39 [aOR = 1.76; CI = 1.01, 3.05] and 40–49 [aOR = 2.48; CI = 1.22, 5.05] also had higher odds of hearing about STIs compared to their younger counterparts aged 18–29 years.

**Table 4:** Factors associated with awareness on sexual and reproductive health, sexually transmitted infections, and HIV/AIDS

Variable	Heard about SRH aOR[95%CI]	Heard about STIs aOR[95%CI]	Heard about HIV /AIDS aOR[95%CI]
Disability type			
Physical disability	Ref.	Ref.	Ref.
Visually impaired	1.139[0.79,1.65]	2.02***[1.39,2.94]	2.32*[1.21,4.44]
Age			
18–29	Ref	Ref	Ref
30–39	1.22[0.67,2.21]	1.76*[1.01,3.05]	2.14[0.88,5.23]
40–49	1.92[0.89,4.17]	2.48*[1.22,5.05]	2.82[0.99,8.00]
50–59	0.98[0.49,1.95]	1.85[0.97,3.50]	1.01[0.67–4.22]
60 and above	1.38[0.66,2.89]	1.35[0.72,2.55]	1.97[0.73,5.32]
Sex			
Female	Ref.	Ref.	Ref.
Male	1.19[0.82,1.74]	0.81[0.55,1.18]	1.09[0.59,2.02]
Residence			
Kumasi Metropolis	Ref.	Ref.	Ref.
Offinso North	1.11[0.64,1.92]	1.22[0.76,1.97]	0.65[0.31,1.35]
Education			
No formal education	Ref.	Ref.	Ref.
Primary	1.37[0.79,2.38]	0.96[0.57,1.61]	0.68[0.33,1.40]
Junior High School	1.65[0.99,2.74]	1.27[0.78,2.08]	1.97[0.82,4.75]
Senior High School/Tertiary	1.40[0.87,2.24]	1.63[0.98,2.71]	1.07[0.51,2.26]
Religion			
Christian	Ref.	Ref.	Ref.
Non-Christian	0.77[0.38,1.56]	1.02[0.55,1.88]	1.19[0.49,2.87]
Marital status			
Never married	Ref.	Ref.	Ref.
Married	1.54[0.91,2.59]	1.19[0.73,1.93]	0.72[0.32,1.61]
Separated/Widowed/Divorced	1.27[0.72,2.24]	1.08[0.61,1.92]	0.65[0.25,1.71]
Ethnicity			
Akan	Ref.	Ref.	Ref.
Non-Akan	1.25[0.64,2.46]	0.81[0.49,1.33]	1.08[0.50,2.33]
Employment			
Not working	Ref.	Ref.	Ref.
Working	1.62*[1.02,2.59]	1.20[0.78,1.86]	1.13[0.55,2.33]
Income (GHC)			
0–99	Ref.	Ref.	Ref.
100–299	1.26[0.79,2.03]	1.25[0.78,1.99]	1.45[0.68,3.07]
300 +	1.01[0.58,1.76]	0.95[0.56,1.63]	1.07[0.45,2.58]
Duration to the nearest health facility			
Less than 30 min	Ref.	Ref.	Ref.
30–59 min	1.02[0.65,1.58]	1.17[0.78,1.73]	0.95[0.45,2.00]
60 min and above	0.85[0.50,1.44]	1.01[0.63,1.61]	0.84[0.37,1.90]
N	402	402	402

*SRH* Sexual and Reproductive health, *STIs *Sexually transmitted infections, *Ref* Reference category; *p < 0.05, **p < 0.01, ***p < 0.001

### Phase 2: qualitative findings

The qualitative component of the research comprised 37 participants (22 males and 15 females), whose ages spanned from 21 to 60 years. Predominantly sourced from the Kumasi Metropolis (n = 24), the participants were primarily affiliated with the Akan ethnic group (n = 31), adhered to the Christian faith (n = 34), and were married (n = 19). The rest of the participants were from the Offinso north district (n = 13) and various other ethnic backgrounds (n = 6).

#### Themes from data

The thematic analysis of the data yielded four overarching themes comprising (a) conceptualisation of SRH, (b) engagement in SRH information seeking, (c) tensions between knowledge and religious beliefs (d) perceived utility of SRH services.

#### Theme one: conceptualisation of sexual and reproductive health

The first theme that emerged from the data was conceptualisation of SRH*.* The issues discussed under this theme comprised PwDs description of what SRH entails and the various types of SRH commodities and services. From the data, both groups of PwDs, males and females from both districts demonstrated good understanding of SRH. They described SRH as involving measures to prevent unintended pregnancies, to prevent and treat STIs, to maintain menstrual hygiene, and to pursue a safe and healthy sexual life in general.

*“In my view, sexual and reproductive health is all about doing things that will protect you from sexually transmitted infections and unintended pregnancies. In terms of safe sex, you don’t have to have sex with every woman you meet. You have to make sure that you know the STI status of anyone you want to have sex with”*. *[Physically Disabled, Offinso North, Male*, *57 years]*

*“I know that sexual and reproductive health is about family planning and contraceptive use so that you can easily space out your children and prevent sexually transmitted infections such as HIV/AIDS”. [Visually Impaired, Offinso North, Male, 31 years]*

Participants also demonstrated knowledge of the various types of SRH commodities, including contraceptives and where to access these services. Furthermore, some participants exhibited awareness of some STIs such as Gonorrhea and Candidiasis.

*“When it comes to the type of family planning, I know about the implant that women insert in their arm. There is this girl here who did that, and I didn’t know. Whenever you hit her arm, you can feel like there is a metallic thing in there. So, she later confirmed that it was an implant. There are others who go for the oral contraceptive pills. I also know about the condom. If it comes to where people can get access to family planning commodities, they can go to the hospital or the pharmacy. For the condom and contraceptive pills, you can easily get it from the pharmacy”. [Physically Disabled, Offinso North, Male, 46 years]*

*“…Also, I am aware that SRH services include treatment and prevention of STIs such as Gonorrhoea. Previously, these diseases were only through sex but now, you can even contract some STIs such as candidiasis from the toilet facilities that we use. If you don’t want to get pregnant or get infected with any STI, then you can use a condom”. [Physically Disabled, Kumasi, Female, 33 years]*

#### Theme two: engagement in sexual and reproductive health information seeking

The second theme that emerged from the data was participants engagement in SRH information seeking. Participants reported various sources from which they obtained information about SRH. These sources included the media, disability organisations, HPs, extended family members, friends, and partners. The most commonly used source was mass media, which encompasses the internet, TV, and radio. For most participants, the media played a vital role in learning about SRH issues. The majority of the visually impaired were active information seekers from the radio.

*“I do research from Google and sometimes too I discuss it with my friends. Also, in school at the JHS and SHS level, they taught us this [SRH]. I also read books; I access Google to get more knowledge or information whenever I need to”. [Visually Impaired, Kumasi, Female, 21 years]*

*“I got this information [SRH] from the doctors who came to serve as facilitators for the health workshop. The union–XXX, invited the doctor from XXX to come and educate us on sexual and reproductive health. This happened in 2020, thus around the COVID-19 time. The time COVID-19 was really serious. Primarily, we get this information on SRH from the healthcare providers who are invited by the XXX. Apart from that, I have my radio where I receive a lot of information. Every Sunday, I tune my radio to listen to [XXX] who is at a government hospital at XXX at 5:30pm. He is a gynaecologist so all that he talks about are SRH and obstetric issues. So, he talks about family planning, birth control and so forth”. [Visually Impaired, Offinso North, Male, 55 years]*

#### Theme three: tensions between knowledge and religious beliefs on sexual and reproductive health services

Another major theme that emerged from the data was tensions between knowledge on SRH services and religious beliefs. Despite having knowledge and awareness of SRH services, some of the participants in both rural and urban areas held various misconceptions regarding the access and utilisation of certain SRH services and products. These misconceptions were exacerbated by their limited comprehensive knowledge of SRH and strong religious beliefs and doctrines opposing the use of contraceptives. Some participants expressed the view that family planning services should only be accessed by those who are married or already have children, and there was a belief that using family planning products could lead to side effects. Others also believed that the use of condoms reduces sexual pleasure.

*“It is something for those who are married. Sometimes, when you are married, you will want to space out your children. That is when the family planning comes through. You can go in for which ever will suit your system so that you can concentrate in life. We all know that sex is not for children, therefore, family planning is not for children”. [Visually Impaired, Offinso North, Female, 25 years]*

*“I think the family planning is good for those who have given birth before because I heard if you haven’t given birth and you do it consistently, it can cause infertility, or you might not be able to give birth”. [Physically Disabled, Kumasi, Female, 27 years]*

Even though most of the participants were aware of SRH services, religious beliefs also influenced their perceptions of some SRH products. Others equated the use of contraceptive services, such as condoms, to abortion. Some participants also discussed the tenets in the Bible regarding the need to give birth to more children to fulfill the responsibility God has imposed on humans to continue the existence of humanity on Earth.

*“…But in my view, it is sinful in the sight of God because using a condom is equivalent to aborting your pregnancy”. [Physically Disabled, Offinso North, Male, 57 years]*

*“…Also, I know about condoms. However, for that one, the Bible speaks against it. The Bible says that we should give birth and fill the earth. So, if you are using condoms, then how do you expect to give birth and fulfil this command by God. So, using condoms is very bad. I know that both family planning and condoms are the same because they both aim to prevent pregnancies. But in my view, if you abstain, that will be much effective at preventing STIs than using the condoms which is against the tenets of the Bible”. [Physically Disabled, Offinso North, Female, 60 years]*

Other participants also indicated how the use of some SRH products diminishes sexual satisfaction.

*“…But I am not a fan of condoms because it is not pleasurable. It is just like eating a toffee with the wrapper on. So, I will never use a condom”. [Physically Disabled, Offinso North, Male, 47 years]*

#### Theme four: perspectives on utility of sexual and reproductive health services

The fourth theme that emerged from the data was perspectives on utility of SRH services. The issues discussed under this theme were importance of SRH services and perception of functionality of SRH services.

##### Importance of accessing sexual and reproductive health services

Five main issues related to the importance of accessing SRH services were discussed. These included STIs prevention, prevention of unintended pregnancies, safe delivery, reduction in maternal mortality, and prevention of economic stress among couples. Most of the participants shared their knowledge on how access to SRH services, such as condoms, can prevent them from contracting STIs, including HIV/AIDS. Participants also demonstrated an understanding of the dual protection that condoms provide, providing protection against both unintended pregnancies and STIs. Some participants further discussed how access to SRH services can help reduce societal stigma.

*“These services help to prevent infections. For instance, using a condom can help prevent infections like syphilis and gonorrhoea. Then with the family planning, it can help us to effectively space out our pregnancies. Also, these sexual and reproductive health services encourage women to go for antennal care until they give birth”. [Visually Impaired, Offinso North, Male, 38 years]*

*“SRH services is good because it helps women to deliver safely. Doctors and health care providers have a lot of expertise and experience to handle pregnancy complications. And so, if you deliver at the hospital, they can easily detect when there is a problem with your pregnancy and provide you with the needed support. When I got pregnant, they [health care providers] assessed my condition and told me I needed to undergo a caesarean section. So, you don’t have to relent on antenatal care because it really helps to ensure safe delivery of your child”. [Physically Disabled, Kumasi, Female,33 years]*

##### Functionality of sexual and reproductive health services

PwDs discussed the functionality of SRH as a continuum under availability, accessibility, and affordability. These issues also reflect the WHO [55] right to health framework. The first issue discussed by the participants regarding the perception of functionality of SRH services was the availability of such services and awareness of value of SRH services. Some of the participants especially the male physically disabled indicated that SRH services were available in various community settings, such as hospitals and pharmacy shops, ensuring accessibility for all community members regardless of their disability status.

*“I know that if I want to use family planning or buy any of the family planning commodities, I can go to the pharmacy shops. If you have an STI, you can go to the pharmacy, and they may have a small room where you can receive an injection to treat the disease. [Physically Disabled, Kumasi, Male*, *36 years]*

*“If you only seek for SRH services, you will get it. It doesn’t matter your disability status. You will be provided family planning if you ask for it. No one can stop you from doing family planning”. [Physically Disabled, Offinso, Male, 46 years]*

*“If I need information about condom or some contraceptives, I can easily move to the pharmacy shop and ask the pharmacist or the attendant there. So, in any drug store, you can get condom to buy. So, a disabled person can also easily go there to get some”. [Physically Disabled Offinso, Male, 50 years]*

##### Accessibility

The second issue that emerged from the participants' discussions on the functionality of SRH services concerned the accessibility of these services. While a few of the participants encompassing all demographic groups perceived that SRH services were accessible, the dominant view among the majority was that these services were inaccessible. The data also showed that, the type of disability and support from abled partners influence level of accessibility.

*“I think that is easy to access SRH services as a PwD. However, for my colleagues who use a wheelchair they have to go to a health facility that is disability-friendly so that they can easily access the facility and services. I use the clutches and so that is not a challenge for me”. [Physically Disabled, Kumasi, Male*, *36 years]*

*“I think to an extent the services are meeting the needs of PwDs. Although I said I got support from my partner, but anytime I attend hospital they take good care of me. And this made me to honour all my ANC appointments. So, it's functional”. [Physically Disabled, Offinso North, Female, 46 years]*

Conversely, PwDs from both rural and urban areas who also felt the SRH are inaccessible shared their views in the following quotes:

*“In some ways, it is okay. But it doesn’t really meet our needs because there are still challenges like how the healthcare providers talk to us. But because the services are available for us to access, we can manage that situation”. [Visually Impaired, Offinso North, Female, 51 years]*

*“I will say no they are not functional because people don’t regard PwDs. It is recently that the education has been intensified. When you go to the health facilities, it is not disability-friendly. If you don’t take care, you may not be able to enter the premises because of how the building is constructed. So, there is still more to be done to make them more functional”. [Visually Impaired, Kumasi, Male, 45 years]*

*“…But the truth of the matter is that PwDs always experience difficulties using some of these services. Talking about the transport system, I mean moving around when you are at the health facility and the nature of the beds. Sometimes you are struggling to climb but you need to force yourself and do it because you are not feeling well. So, I will say, they are not fully functional when you factor PwDs into the equation”. [Physical Disabled, Offinso North, Female, 60 years]*

##### Affordability

The third sub issue that emerged from the participants regarding the functionality of SRH was the affordability of the services. Although participants shared two contrasting views on the affordability of these SRH services, the prevalent view was that the high direct and indirect costs associated with accessing them were a barrier. The main challenge associated with this was the ineffectiveness of the NHIS, which makes it difficult for patients to obtain subsidised medications. The data also revealed that some SRH providers are friendly and supportive of the PwDs. Those who perceived the services to be affordable shared the following views:

*“I remember that during my postnatal care visit to the hospital, we were given the option to choose any of the family planning methods and it will be provided to us for free”. [Visually Impaired, Offinso North, Female, 33 years]*

*“I was shy though, but the attendant was kind to me and did not discriminate against me. So, it was easy for me to get it. Moreover, the cost of the condom was not expensive for me to bear”. [Physically Disabled, Offinso North, Male, 53 years]*

However, the majority of PwDs irrespective of type of disability who expressed opposite views on the affordability of the SRH services stated the following opinions:

*“The challenge is that some of the most major diseases are not covered by the national health insurance. Though it is there but sometimes when you are sick and needs certain medications that are not covered you must still use money to get those medicines. It just covers some minor medications. And this is a challenge because if they want it to really take care of the very poor then it should cover most of the diseases that are costly but when you go there, they will just give you some basic medications”. [Visually Impaired, Kumasi, Male, 25 years]*

*“Also, the distance to the hospital and the amount of money that you might end up paying at the hospital can be a disincentive for you to go to the health facility. When you are going to deliver at the hospital, you have to buy a lot of things. These things are very expensive. So, these things are major challenges especially when you don’t have money or people to support you financially”. [Physically Disabled, Kumasi, Female, 33 years]*

### Triangulation of study findings

Table 5 provides a summary of the integration and synthesis of the qualitative interview findings and quantitative survey results.

**Table 5:** Triangulation of study findings

Theme	Issues discussed	Quantitative finding	Illustrative qualitative quote	Synthesis
Conceptualisation of SRH	Types of SRH services and STIs	The majority of the respondents indicated that they have ever heard about SRH (94.3%), Sexually transmitted infections (92.5%) and HIV/AIDS (97%)	*“I also know that SRH is also about having a healthy sexual life. There are some people who engage in multiple sexual partnerships even when they are married. You don’t know the kind of disease the person has. So, if you are not lucky, you can contract diseases like gonorrhoea and HIV”. [Physically Disabled, Kumasi, Male, 56 years]*	This indicates that the majority of the respondents were aware of the concept of SRH, STIs, and HIV/AIDS. However, there are still misconceptions surrounding some of the SRH commodities. This calls for tailored education to address these misconceptions
Active engagement in SRH information seeking	Sources of information on SRH services	Radio/TV was the most common source of information on SRH, reported by 91.4% of the respondents 68.8% indicated that health workers was their source of SRH information	*“I normally listen to the radio. So, whenever I hear anything about SRH being discussed on radio, I will tune in to it and listen. Usually, I listen to XXX FM every Saturday being 12:30 pm and 2:30 pm. They have a health show called “you and your health”. They invite nurses to come on board to explain such issues to us. XXX too has a similar programme on health. They discuss adolescent issues and sexually transmitted infections. They also talk about personal hygiene and menstrual hygiene. Personally, I organised a programme on community water, sanitation, and hygiene. Because of my condition, I don’t normally go to the internet. Growing up, we were not well-versed in ICT. We were not even having phones. It was the radio station that was serving us. So, I rely on the radio. If not, I have to rely on my children to read out information to me because I cannot read for myself. It is the radio stations who are helping”. [Visually Impaired, Kumasi, Male, 41 years]*	Radio and television were the major sources of SRH information among PwDs. Some of the PwDs also indicated that their source of SRH information was healthcare providers. This represents a great opportunity for qualified individuals to provide appropriate education on SRH to persons with disabilities. This could also help reduce misconceptions surrounding some SRH products
Tensions between knowledge and religious beliefs			*“I heard it, but I have one friend who did family planning, she put something in the arm, I think for 5 years but I think she has removed it now. I advised her to remove it because you know that as a lady you have to get your monthly flow if only you are not pregnant and because of that she doesn’t get her monthly flow, maybe if she gets it this month, it takes 3 months before she gets another one. So, what is the essence of putting it there? Just protect yourself but don’t use that, me I will not advise them to use that thing. It can cause fibroid, if you are not able to get your monthly flow it can cause fibroid. Because it is something that it is dirty, so that is why every month it has to come out, if the egg is not fertilized then it has to turn to blood and come out so if you are avoiding it to be fertilized and it is not coming out, what do you expect? It can be like blood clot so when it happens like that it can cause fibroid. And fibroid too is a serious condition among females”. [Visually Impaired, Kumasi, Female, 25 years]* *“I see it as a sin to be practicing family planning. Plus, I have seen people who have used it and then they put on a lot of weight. Because I am already overweight, I would not want to add more weight. Also, I have heard people say that when you do family planning, it affects your menstrual cycle and leads to heavily bleeding”. [Physically Disabled, Offinso, Female, 36 years]*	Although the awareness level is high, there are still some inherent misconceptions and religious beliefs that need to be addressed in order to increase the utilization of sexual and reproductive health services
Perceived utility of SRH services	STIs prevention	– 95% indicated that condom use is a main strategy to prevent STIs – The most frequently reported importance was getting educated about STIs (98.9%)	*“Also, if you use the condom, you will protect yourself from Gonorrhoea and other STIs. Mostly, a lot of beautiful women have STIs because a lot of men have sex with them”. [Physically disabled, Kumasi, Male, 36 years]*	This shows that the majority of the respondents were knowledgeable about the use of condoms and their effectiveness in preventing STIs
	Prevent unintended pregnancy	– 93.4% indicated that access to contraceptives is an important aspect of SRH services and this could prevent unintended pregnancy	*“Sexual and reproductive health information helps me delay childbirth or prevent me from getting pregnant so that I can have time to finish the seamstress work because I just started. When you space your birth, it helps you to be emotionally stable and stress free because when the children are many it makes it difficult for the parents to take care of the children when the parents are poor”. [Physically disabled, Kumasi, Female, 27 years]*	Preventing unintended pregnancies is a key strategy for many women to achieve their economic aspirations. It is also a means to empower women with disabilities and promote their economic wellbeing which invariably improves their overall quality of life
	Safe delivery		*“Also, through SRH services, women are advised to seek medical care from the hospital when they are pregnant. That alone helps them have safe delivery. I remember one of my sisters delivered at home and she ended up getting complications. But because the birth attendant was an old woman, she did not know what to do. At the end of the day, we rushed her to the hospital, but she died on arrival at the facility. So, from that experience, I learnt that it is important to always ensure that the women deliver at the hospital”* *[Physically Disabled, Kumasi, Male, 47 years]*	The utilization of sexual and reproductive health services such as antenatal care, health facility delivery, and postnatal care plays a crucial role in improving maternal health outcomes
	Perceived functionality of SRH services	– The overall functionality score was 15 ± 4.9 – The visually impaired (16.6 ± 5.0, *p* < 0.001), those with Senior High School/Tertiary level of education (16.7 ± 5.3, *p* < 0.001) and those who earn GHC300 or more (14.6 ± 4.9, *p* < 0.001) perceived SRH to be unfunctional	*“If you only seek for SRH services, you will get it. It doesn’t matter your disability status. You will be provided family planning if you ask for it. No one can stop you from doing family planning”. [Physically Disabled, Offinso, 46 years]*	This shows that the ability of PwDs to access SRH services depends on their level of self-efficacy. Additionally, perceptions of the functionality of the health system vary based on the demographic characteristics of PwDs. However, not all PwDs are sufficiently empowered to easily seek SRH services. Therefore, it is important to enhance the functionality of the health system
			*“I don’t think it is functioning effectively. The last time, I was going to renew my health insurance, but I never knew that renewal of health insurance was free for PwDs until one of our members in the disability association informed me. So just imagine”. [Physically Disabled, Kumasi, Male, 47 years]* *“…But I am sceptical about the extent to which the SRH services meet our needs as PwDs. I know my rights and so, I will always insist that you go by that. But for those who don’t know their rights, it is easy for them to face challenges”. Physically Disabled, Kumasi, Male, 56 years]*	Most of the respondents perceived the health system as dysfunctional in meeting their sexual and reproductive health needs. This calls for a multisectoral approach to make the health system more user-friendly, thereby increasing the accessibility of SRH services for PwDs

## Discussion

This mixed-methods study aimed to assess the awareness and knowledge of PwDs regarding SRH and their perceptions of the functionality of SRH services in Ghana's Ashanti Region. The findings indicated that most participants were aware of SRH (94.3%), STIs (92.5%), and HIV/AIDS (97.0%). The findings also revealed that employment status, type of disability, and age group were associated with different levels of awareness of SRH, STIs and HIV/AIDS. Moreover, the study showed that PwDs generally perceived SRH services as dysfunctional in meeting their needs. The perception of the functionality of SRH services varied significantly by type of disability, level of education, and income level. The qualitative data complemented and enriched the quantitative data by providing insights into the meanings, misconceptions, and beliefs that PwDs had about SRH services.

The qualitative data revealed that PwDs in both rural and urban areas understood SRH as encompassing various aspects, such as preventing unintended pregnancies, preventing, and managing STIs, and enjoying a safe and healthy sexual life. These meanings are in line with previous studies conducted in Ghana [33] and Vietnam [21]. Both the quantitative and qualitative data indicated that PwDs had a high level of awareness and knowledge of HIV/AIDS. The quantitative data showed that 97.0% of PwDs had heard about HIV/AIDS, which is higher than the rates reported in previous studies in Southern Ethiopia [24], Addis Ababa [56], and North-Shewa zone, Ethiopia [19]. Similarly, the quantitative data revealed that 92.5% of PwDs had heard about STIs, which is also higher than the prevalence reported in North-Shewa zone, Ethiopia [20]. When combining these findings under the broader category of SRH awareness, which was about 94.3% in this study, the level of SRH awareness is higher than the levels reported in previous studies among adolescents with disabilities in Ghana [23] and young PwDs in Ethiopia [56].

The differences in the levels of awareness observed in this study compared to others could be attributed to several factors. First, access to SRH services may vary across different settings, as suggested by Mekonnen et al. [19]. Second, socio-demographic and socio-cultural characteristics of the participants may influence their exposure to SRH information, although this current study considered two groups of disability from both rural and urban areas. Third, the modes and sources of dissemination of information on STIs, HIV/AIDS, and SRH may affect the awareness levels of PwDs. Fourth, the degree of attention given to SRH issues affecting PwDs in each study area or country may differ. Fifth, the type or form of disability among respondents may determine their level of exposure to SRH information and overall access, as highlighted by Kassa et al. [56] and Mekonnen et al. [24] in Ethiopia. For example, PwDs who are visually or hearing impaired may have different levels of exposure to SRH information through mass media than those who are mobility impaired. In this present study, the visually impaired in both rural and urban areas resorted to the radio for information on SRH services.

The framework guiding this study also suggests that client characteristics influence various health outcomes [35–37]. In line with a previous study in Ethiopia [24], this study found that employed PwDs were more likely to hear about SRH than those who were not working. The association between employment status and health has been well established in the literature, with evidence suggesting that good health is positively related to active employment [57]. Employed PwDs may have economic and financial advantages over those who are not working in terms of accessing SRH services and subscribing to mass media [58]. It is also possible that those who are working are more likely to be literate and literacy has a positive correlation with improved access to information [5].

Consistent with previous studies in Ghana [23] and China [59], visually impaired persons were more aware and knowledgeable about SRH, including STIs and HIV/AIDS, than persons with other types of disability. Badu et al. [34] also reported that visually impaired women were active seekers of SRH information. However, the generalisability of this findings to support this study should be done cautiously, as the study by Badu et al.[34] only sampled visually impaired women. This study finding on the association between type of disability and knowledge of SRH such as STIs and HIV/AIDS is also supported by a previous study among young PwDs in Ethiopia [56]. A possible explanation for this finding is that other disabilities, such as hearing impairment, face more challenges accessing SRH information than those with visual impairment mainly due to difficulties in verbal communication and comprehension from reading [59]. The qualitative data also revealed that most visually impaired PwDs actively sought SRH information from the radio. It has also been suggested that visually impaired persons are more able to socialize and learn from people than their counterparts with other types of disability, such as those with hearing or mobility impairment [60].

Age was also significantly associated with knowledge and awareness of STIs in this study. PwDs aged 30–49 years were more likely to hear about STIs than their younger counterparts aged 18–29 years. This finding on the association between age and knowledge of STIs is in line with a previous study in Ghana [23]. One reason for this could be that older age implies more exposure to SRH information due to longer life span. Older age could also imply more exposure or more years of education, especially as many PwDs get their SRH information from friends, family members, and/schoolteachers [23, 25, 34].

Another major theme that emerged from the data was tensions between knowledge and religious beliefs about SRH services among some participants across all demographics. Despite the knowledge and awareness on SRH services, some of the participants had various misconceptions about the access and use of some SRH services and products. These misconceptions were influenced by religious beliefs and doctrines that opposed the use of contraceptives. The influence of religion on SRH and health seeking behavior of PwDs has also been reported in previous studies in Ghana [9, 32]. Some of the participants believed that family planning services should only be accessed by married people or those with children, and that using family planning products could contribute to dizziness, infertility, fibroid and obesity. This finding is consistent with existing literature in Eastern Nepal [61] and Northwestern Tanzania [62]. In the Nepal study, some respondents stated that their holy scriptures did not allow them to use family planning or any SRH method because they believed that children are gifts from God and any artificial barrier to this blessing (pregnancy or childbirth) is a religious offense [61]. Nketsia et al. [9] also found that religious affiliation influenced deaf people’s awareness of pregnancy and abortion services. They further argued that any SRH intervention lacking consideration for the influence of religion and culture was likely to be ineffective. Consequently, new policy initiatives addressing SRH ought to prioritize establishing a robust collaboration between policymakers and religious institutions to promote awareness campaigns and educational programs specifically targeting PwDs to dispel and clarify many of the misconceptions influenced by religion and culture in Ghana.

Regarding the functionality of SRH services, most PwDs who perceived SRH as dysfunctional interpreting it as not meeting their needs. In accordance with the health outcomes model [35–37], the clients characteristics influenced the perception of the functionality of SRH services. Specifically, the visually impaired, those with senior high school/higher level of education and those who earn GHC300 or more rated the SRH as dysfunctional. The qualitative data showed that the functionality of SRH were viewed as a continuum with availability, accessibility and affordability as the key issues discussed. For instance, most of the participants, especially the physically disabled expressed issues with the accessibility of infrastructure, while others mentioned negative attitude of healthcare providers. Most of the participants expressed that family caregivers often emerge as the sole resources capable of helping them overcome barriers encountered when seeking SRH services. The lack of support from family caregivers, therefore, highlights the potential frustration experienced by PwDs at SRH service facilities and care centers, mainly due to the unique requirements and characteristics associated with their disabilities [41]. This finding is consistent with studies conducted in Uganda [63, 64] and SSA [65]. These accessibility issues expressed by participants of this study are also similar to those reported by the WHO [5] as key accessibility barriers to SRH by PwDs. The infrastructural inaccessibility of SRH facilities expressed by participants is an indication of the continuous marginalisation of PwDs in Ghana, as they are often neglected or excluded in the infrastructural development. This is also in violation of the “Persons With Disability Act, Act 715 of Ghana” [28] which aims to promote the wellbeing, dignity and quality of life of PwD as enshrined in many human rights and international conventions that Ghana has signed [66]. Strict enforcement of the Act and more education and sensitization are needed to empower PwDs to demand their rights. SRH service providers need better and improved training to foster improved relations with PwDs to make SRH services friendly and functional to PwDs.

## Implications for policy and practice

The findings of this study have various policy and practice implications. First, SDGs 3.7 and 5.6 strive to achieve universal access to SRH information and services as well as the integration of SRH into national strategies and the realisation of universal access to SRH and rights, respectively [67]. Although PwDs had high awareness and knowledge on SRH, some still held religious doctrines and misconceptions about SRH services. To maintain the knowledge and further improve it, tailored educational campaigns to dispel the myths surrounding some SRH products is required by the HPs. These educational campaigns could be delivered via radio and television. Second, some PwDs identified HPs as a valuable source of SRH information. This presents an important opportunity for adequately trained professionals to deliver targeted SRH education to PwDs. By doing so, misconceptions surrounding certain SRH products can be effectively addressed and mitigated. Third, the utilisation of SRH information by a subset of women facilitated the prevention of unwanted pregnancies, thereby serving as a strategy for realizing their economic aspirations. Moreover, this practice represents a mechanism through which women with disabilities can be empowered, fostering their economic empowerment in the process. Fourth, the capacity of PwDs to access SRH services depends on their level of self-efficacy [41]. However, not all PwDs have the necessary empowerment to readily access SRH services. This calls for measures to empower PwDs. Furthermore, the perception of the health system’s functionality in meeting SRH needs varies among PwDs, influenced by their distinct demographic attributes. Therefore, it is essential to improve the functionality of the health system to effectively address these challenges.

## Strength and limitations of the study

The strength and limitations inherent in this study are worth discussing. The strength of this study lies in the use of a sequential explanatory mixed-methods design, which enabled the integration of both quantitative and qualitative research findings [43]. This approach enhanced the comprehensiveness and depth of the study, allowing for a more detailed understanding of the PwDs’ awareness and knowledge of SRH services and their perception of the functionality of these services. The study also sampled respondents from both rural and urban areas, making it possible to generalize the findings to these settings. Despite this, the study focused on only two categories of disabilities: those with visual impairments and those with physical disabilities. In addition, self-reported data was collected from these participants, hence, social desirability and recall biases are possible. However, triangulation of the study findings helped to ameliorate this limitation.

## Conclusions

In conclusion, the study found relatively high awareness and knowledge about SRH services among PwDs. Type of disability was associated with awareness of STIs including HIV/AIDS. However, some misconceptions influenced by religious beliefs about SRH services persist. PwDs also perceived SRH service delivery as largely dysfunctional. The findings call for tailored education to reduce misconceptions and put in pragmatic steps to deliver quality SRH services and information to PwDs. Further studies could explore the sexual lives of PwDs and recommendations from stakeholders on the strategies to improve SRH outcomes among PwDs in Ghana.

### Supplementary Information

**Additional file 1.** Questionnaires for_PWDs.

**Additional file 2. **STROBE_Checklist.

**Additional file 3.** IDI_Guide_PWDs.

**Additional file 4.** COREQ.

## Data Availability

The dataset is available upon reasonable request from the corresponding authors (A.S and T.I.E).

## Abbreviations

aOR: Adjusted odds ratio
CI: Confidence interval
COREQ: Consolidated criteria for reporting qualitative research
GHS: Ghana Health Service
GSS: Ghana Statistical Service
JCU: James Cook University
KATH: Komfo Anokye Teaching Hospital
NHIS: National Health Insurance Scheme
PwDs: Persons with disabilities
RAs: Research assistants
SRH: Sexual and reproductive health
UN: United Nations
HPs: Healthcare providers
WHO: World Health Organization
