# Supplementary material for: A mixed-methods study of the awareness and functionality of sexual and reproductive health services among persons with disability in Ghana
Source: Reprod Health. 2023 Oct 31;20:162. doi: 10.1186/s12978-023-01700-1 (PMC10619320; doi:10.1186/s12978-023-01700-1)
Supplement: Supplementary file 3 — Additional file 3. IDI_Guide_PWDs. [file 12978_2023_1700_MOESM3_ESM.docx]

## IN-DEPTH INTERVIEW GUIDE FOR PWDs

**SECTION 1: CLIENT CHARACTERISTICS**

1. Please tell me about yourself (Age, Occupation, Monthly income, Disability duration, Educational level, Marital status, Number of children, Religion and Ethnicity).

**SECTION 2: KNOWLEDGE AND SOURCES OF INFORMATION ABOUT SEXUAL AND REPRODUCTIVE HEALTH SERVICES AND CARE**

What is your understanding of Sexual and Reproductive Health (SRH)? (Probe for STIs prevention, family planning and types of contraceptives, safe delivery, safe sex)

How did you gain this understanding/ obtain this information?

In your opinion, what is the importance of accessing SRH services?(probe for improving SRH, reducing STIs, birth spacing, safe delivery, reduce deaths)

1. How do you access SRH information? (Probe for sources of SRH information–radio, TV, friends, friends, NGOs, Disability organization, etc)
2. What is the content of SRH information (probe for STI prevention, personal hygiene, menstrual hygiene)
3. What are the challenges and barriers in accessing SRH information (probe the format of the information, language, not able to hear/see/move, unable to understand).

How do you perceive the functionality of SRH services and delivery in Ghana?

**SECTION 3: SEXUAL BEHAVIOUR OF PWDS AND SRH OUTCOMES**

1. Tell me about any sexual relationships you have ever had (probe for ever had sex, age at first sex, duration of sexual relations/marriage, number of sexual partners).
2. Can you tell me about your experience of any gender-based violence?
   1. Physical violence[punch, twist arm, threaten to hurt] (probe for cause, perpetrator, number of times, action taken/help sought, social support received).
   2. Sexual violence[force/perform sex when you didn’t want to] (probe for cause, perpetrator, number of times, rape, sexual coercion, action taken/help sought, social support received).
   3. Emotional violence [insult, say something to hurt you] (probe cause, perpetrator, number of times, insult based on disability, action taken/help sought, social support received), exclusion from social events due to disability
   4. Pregnancy termination (Probe reason for termination–choice or triggered termination, mode of termination).
3. How will you describe your sexual and reproductive health status (Probe for STIs, ever checked for HIV status, rating of SRH status, Menstrual problems among women with disabilities and management )

**SECTION 4: ADOPTION AND UPTAKE OF SRH SERVICES**

1. Please tell me about any SRH intervention/policy you have heard about (Probe for source)
2. Which SRH interventions/Policies are for PWDs
   1. Probe for National health insurance scheme, LEAP, Disability Act, Free maternal health care)
3. Which of these interventions have you ever accessed?
   1. Probe for number of times, reasons for the uptake and enablers for uptake
   2. Challenges in accessing each of the interventions (probe for distance, Delay in release of funds, inability to access, lack of information about policy/intervention, cost, ﻿unfriendly healthcare infrastructure, communication problems, limited support from HPs, mobility problems, poor healthcare services and individual barriers such as lack of knowledge/ confidence etc.)
4. How do you perceive the attitude of health professionals in the process of accessing the SRH services (probe for stigma, discrimination, special treatment, use of derogatory words)?
5. How effective are the SRH interventions in improving your SRH?

**SECTION 5: RECOMMENDATIONS TO IMPROVE THE IMPLEMENTATION OF INTERVENTIONS AIMED AT IMPROVING SRH OF PWDS**

1. ﻿How can your access to and utilisation of SRH interventions and services be improved?
2. END Is there anything else you’d like to share with me today?

**Thank you for your cooperation!**
